# Molecular epidemiology of quinolon resistant strains of extended spectrum beta-lactamase producing *Escherichia coli*

**DOI:** 10.12669/pjms.315.8186

**Published:** 2015

**Authors:** Suleyman Durmaz, Duygu Percin, Baris Derya Ercal

**Affiliations:** 1 Suleyman Durmaz, Konya Numune Hospital, Microbiology Laboratory, Konya, Turkey; 2 Duygu Percin, Erciyes University Faculty of Medicine, Department of Medical Microbiology, Kayseri, Turkey; 3 Baris Derya Ercal, Erciyes University Faculty of Medicine, Department of Medical Microbiology, Kayseri, Turkey

**Keywords:** Epidemiology, ESBL producing *E.coli*, Quinolone resistance, Clonal relationship, rep-PCR

## Abstract

**Objective:**

To determine the clonal relationship of ESBL-producing and quinolone resistant *E.coli* strains and to investigate the risk factors for infections with these microorganisms.

**Methods:**

A total of 95 ESBL-producing and quinolone resistant *E.coli* strains isolated from various clinical specimens of inpatients and outpatients in our hospital were included in the study. Risk factors for infections with ESBL-producing *E.coli* and demographic data of the patients were obtained from hospital records. The rep-PCR method was used for the determination of the genetic relationship of the strains.

**Results:**

Of the strains included in the study, 33(34.7%) were isolated from inpatients and 62(65.3%) from outpatients. At least one risk factor has been identified in all patients for infection with ESBL producing *E.coli* and the mean of the risk factors of patients was 4.2. The most common risk factor was urinary catheter insertion (57.9%). The distribution of the strains in each clone was as fallows: clone A: 9(9.5%), clone B: 10(10.5%), clone C: 38(40%), clone D: 12(12.5%), clone E: 6(6.3%), clone F: 7(7.3%) and clone G 5(5.3%). The clones A, D and C (dominant clone) were isolated from hospital and community acquired infections. Clones E, F and G were identified as nosocomial clones.

**Conclusion:**

Infections with multidrug resistant bacteria may be related to the hospital although they were isolated from outpatients. Developing a medical record system is vitally important to prevent the occurence and spread of resistant bacterial infections in the community.

## INTRODUCTION

*Escherichia coli* emerges as an agent in 70-95% of community-acquired urinary tract infections andin 50% of hospital-acquired cases.^1^ The most important and common cause of resistance to beta-lactam antibiotics preferred to treat these infections is the production of beta-lactamase and especially extended-spectrum beta-lactamase (ESBL). Transition of ESBL production between bacteria and plasmids and transferring along the genes that cause resistance to other antibiotics lead to multiple drug resistance and treatment difficulties.^2^,^3^ On this topic, many studies investigating the presence of ESBL-producing bacteria in the stool of asymptomatic hospitalized patientsand outpatient and defining the risk factors have been conducted.^4^-^6^ The risk factor most emphasized and demonstrated its importance is the use of antibiotics such as fluoroquinolones, trimethoprim/sulfamethoxazole, especially broad spectrum cephalosporins.^7^,^8^ Furthermore, it has been shown in the studies that there were common risk factors for the spread of ESBL-producing strains such as long hostipal stay, to be 65 years of age/premature, catheter insertion to central vein/artery, surgical intervention for intra-abdominal region or urinary tract, the insertion of urinary catheter, diabetic/hemodialysis patients, malignant diseases and urinary tract infections.^5^,^6^,^9^

Studies have shown that the quinolone resistance in ESBL-producing bacteria was 10 to 20 times higher. Chen et al.^10^ have found the quinolone resistance in strains which do not produce ESBL and ESBL-producingstrains as 26.7%, and 84.6%, respectively in their multicenter study. In a study conducted in Italy, the quinolone resistance was found to be 92% and 41.7% in *E.coli* strains which ESBL-producing and not produce.^11^ In our hospital, the quinolone resistance was found to be 82% and 29% in *E.coli* strains which ESBL-producing and not produce.^12^ To investigate the clonal relationship between ESBL-producing and quinolone-resistant *E.coli* strains will be extremely useful for both detection of infection sources and minimizing the economic losses due to infections.

In this study, it was aimed to investigate the risk factors for infections occuring due to ESBL-producing and quinolone resistant *E.coli* strains and to determine the clonal relationship of strains with repetitive-sequence-based polymerase chain reaction (rep-PCR) method.

## METHODS

The permission from ethics committee of Erciyes University Faculty of Medicine, Clinical Research Ethics Review Commission (Decision No. 2010/07) was obtained in order to conduct the study. A total of 95 ESBL-producing and quinolone resistant *E.coli* strains isolated from various clinical specimens of inpatients and outpatients admitted to Erciyes University Hospital between 2010-2011 and stored at -70°C were included in the study. One strain from each patient were included in the study. Strains were identified by routine microbiological methods (colony morphology, gram staining and biochemical tests: motility, indole, lactose/glucose fermentation, methyl red, citrate, urease, hydrogen sulfide, and gas production) and Phoenix 100 (Becton Dickinson, USA) automatized culture system. Quinolone resistance of strains was evaluated by using ciprofloxacin disk with the disk diffusion method, and ESBL production was evaluated by performing double-disk synergy test. Infections caused by these isolates were classified as hospital or community based depending on the medical records of the patients and Centers for Disease Control (CDC) criteria. According to CDC criteria, if infection occurred 72 hours after the hospitalization or within the first 10 days after discharge, it was evaluated as hospital-acquired infections (HAI), if not, it was evaluated as community-acquired infections (CAI).^13^ The patients’ data were examined, and risk factors that could predispose them to ESBL-producing *E. coli* infection were recorded. In many studies conducted by examining the patients’ records, the conditions, which were identified as risk factors associated with the spread of ESBL-producing strains, such as being older than 65 years of age, having malignant disease or cardiovascular disease, being diabetic or hemodialysis patient, the presence of urinary tract disease (benign prostatic hypertrophy, vesicoureteral reflux, ureteropelvic junction obstruction, kidney stones, neurogenic bladder), stay in hospital or intensive care unit (ICU) more than 11 days in the last 6 months, implementation of intubation or mechanical ventilation in the hospital, the insertion of catheter into central vein or artery, surgical intervention for intra-abdominal region or urinary tract, the insertion of urinary catheter, gastrointestinal intervention (biliary drainage tube, jejunostomy tube, etc.), having decubitus ulcer, the use of quinolone or beta-lactam type antibiotics in the last 3 months, having 3 or more urinary tract infections in the last one year, being premature were investigated.^4^-^6^,^9^

The rep-PCR (Diversilab, Biomerieux, France) method was used in order todetermine the genetic relationship between quinolone resistant ESBL-producing *E.coli* strains. Diversilab software Pearson’s correlation coefficient and UPGMA (Unweighted Pairvis Grouping Matematical Avenaging); the grouping method of the unweighted pairs with the mathematical average was used to compare the rep-PCR profiles automatically in making the similarity calculations between isolates. The strains, which weresimilar to each otherover 90%, were regarded as the main clone. Similar clones over 95% in the main clones were considered as subclones. The strains with similarity rates less than 90% were considered as different clones.

## RESULTS

The strains included in the study, 33(34.7%) strains were isolated from hospital-acquired infections and 62(65.3%) strains were isolated from community-acquired infections.

Twenty-one (22%) and 74 (78%) of the patients that the strains included in the study were isolated were children and adult, respectively. The average age of all patients, was found to be 51.2(± 25.9). According to the CDC criteria, 33(34.7%) and 62(65.3%) of the strains included in the study were isolated from hospital-acquired infections (HAI) and community-acquired infections (HAI), respectively. However, when the risk factors were examined, at least one risk factor was determined in all patients. The mean level of the risk factors in patients was found as 4.2. The mean levels of the risk factors in patients with HAI and CAI were 4.6 and 4, respectively (Table-I).

**Table-I:** The presence of risk factors for ESBL-producing E.coli infection.

Risk Factors	All patients n (%)
65 Age ↑	38 (40)
Long hospital stay	37 (38.9)
Prolonged hospitalization at ICU	17 (17.9)
Antibiotic use	34 (35.8)
The presence of malignant disease	32 (33.7)
Urinary abnormalities	24 (25.2)
Cardiological diseases	14 (14.7)
Diabetes mellitus	13 (13.6)
Malnutrition	11 (11.5)
Recurrent urinary tract infections	9 (9.4)
Urinary catheter	55 (57.9)
Surgery of the urinary site	30 (31.5)
Intra-abdominal surgery	8 (8.4)
A-V catheter	23 (24.2)
Gastrointestinal catheter	19 (20)
Intubation	16 (16.8)
Mechanical ventilation	8 (8.4)
Hemodialysis patients	6 (6.3)
The presence of decubitus ulcers	3 (3.1)
Premature birth	2 (2.1)

**Fig.1:**
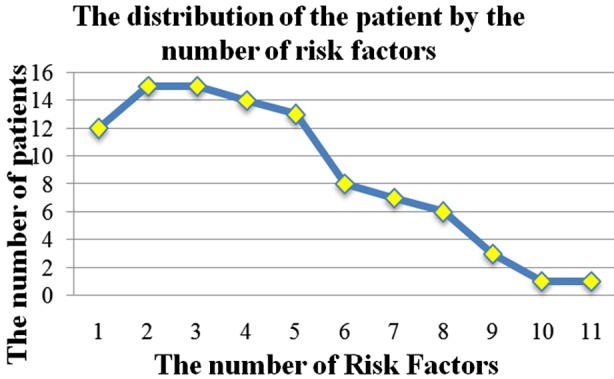
The graph that shows how many risk factors all patients have.

In the study, the most common risk factor was urinary catheter insertion and it was seen in 55(57.9%) patients. Thirty-eight of the patients (40%) were over the age of 65 years, when adult patients were considered, 51.3% of them had risk factor for age. The first three risk factors in patients with HAI were the insertion of urinary catheter in 20 patients (60.6%), being over 65 years of age in13 patients and stay in the ICU in 12 patients. The first three risk factors in patients with CAI werethe insertion of urinary catheter, being over 65 years of age and the presence of malignant disease. It has been shown in Fig.1 that how many risk factor the patients had together.

**Fig.2:**
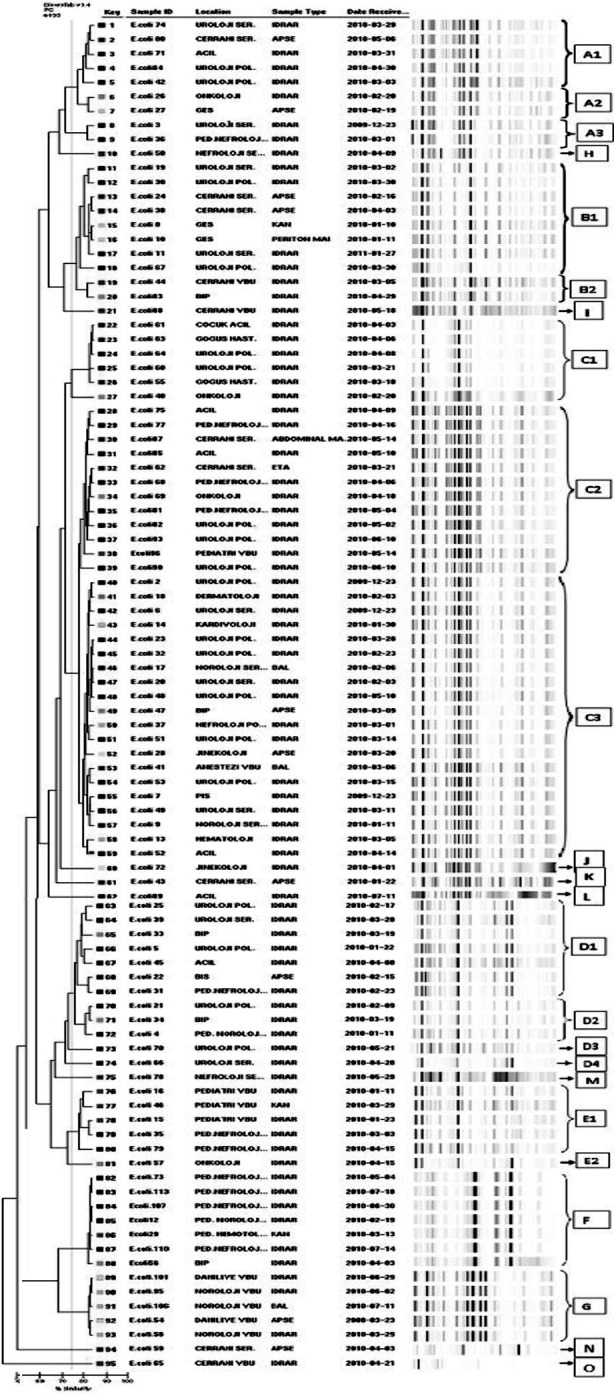
Dendrogram of all strains.

A total of 15 clones (A-O) including 7 (A-G) main clones and 8 sporadic cases were determined in total of 95 *E.coli* strains included in the study. It has been determined that there were 9(9.5%) strains in clone A, 10(10.5%) strains in B, 38(40%) strains in C, 12(12.5%) strains in D, 7(7.3%) strains in F, and 5(5.3%) strains in G. The dendrogram ofthe *E.coli* strain was shown in Fig.2.

According to diversilab rep-PCR results, 9 of 95 ESBL-producing quinolone-resistant *E.coli* strains belonged to clone A, the first strain of this clone was isolated from a patient’s urine which was sent from the urology service. The final isolated strain was isolated from a patient’s urine which was sent from urology outpatient clinic. Although 6 strains ofclone A were isolated from outpatients, these strains were thought to be associated with the hospital due to the hospitalization history in the last 6 months in these patients and having with 4 risk factors as an average for infection with ESBL-producing *E.coli*.

It has been seen that 8 of 10 strains and 2 of them identified in clone B belonged to B1 and B2 subclones, respectively. The first strain of this clone belonged to B1 subclone and was isolated from a blood culture of a patient hospitalized in the internal medicine service. Isolating of the other strain of subclone B1 with an interval of one day from the same service was considered as the transition between patients.

In the study, 38(40%) strains were in clone C which was determined the dominant clone of our study. The 27(71%) strainsof clone C belonged to outpatients. However, the community-acquired strains has been assessed to be associated with the hospital due to the patients had four risk factors as an average for the infection with ESBL-producing, 15 were urological patients and 8 patients except them had a history of urinary tract surgery in the last 3 months.

Nine of 12 strains of clone D were isolated from CAI. However, this clone was considered as hospital-acquired due to 6 of the patients had malignancy, the presence of benign prostatic hyperplasia (BPH) in 2 of them and having 4.4risk factors as an average for the infection with ESBL-producing.

The 5 of the 6 strains and one of them in the clone E were determined as E1 and E2. This clone was considered as hospital-acquired belonging to pediatric hospital building due to all of the E1 subclone were isolated from pediatric patients.

All strains in the clone F were considered as indistinguishable, the first strain of this clone was isolated from an urine culture sent from pediatric nephrology and the last strain was isolated from a patient from the same department. Therefore it was considered as hospital-acquired although it did not cause an outbreak.

Five strains in the clone G were considered as indistinguishable and considered as hospital-acquired due to all strains of this clone were isolated from the patients hospitalized in the internal ICU between March and June.

## DISCUSSION

The coexistence of ESBL production and fluoroquinolone resistance can be explained by the frequent use of fluoroquinolones and beta-lactam agents in humans and food products and the transfer of patient to patient with existence of the multiple resistance gene in the plasmids carrying genes encoding ESBL. Data supporting this are isolation of quinolone-resistant *E.coli* in in poultry and pork in a study conducted in Spain and to be seen plasmid-mediated resistance which was combined with ESBL production in China although the quinolone use in children was forbidden. It has been reported that 470 thousand tons of antibiotics were used in a year in animal feed in China.^8^,^14^ The most common risk factor in our study is the insertion of the urinary catheter. The history of antibiotic use was available in 35.8% of all patients and it was the 4th most common risk factor. It was considered that this situation was caused since we could not reach the records outside of our hospital. Because it has been shown in the studies that both beta-lactams and quinolones were easily and commonly prescribed in the first-level health care facilities in our country.^15^,^16^ In addition, it is seen that the use of antibiotics may be beyond the records that we could reach when considering the patient can buy these antibiotics from pharmacies without the consent of doctors.

Besides the knowledge of the risk factors for infection, the detection of the source, the determination of the relationship between the community and hospital-acquired strains and the development of fighting methods against the resistant bacteria will be useful in ensuring success. For example, while the proportion of ESBL-producing strains in northern France in 1996 was 19.7%, this rate decreased to7.9% in 2000 with surveillance activities and control measures andwas reported to range between 1-5%in later years.^17^

Sidjabat et al.^18^ have investigated the clonal relationship of 49 cephalosporins and quinolone-resistant *E.coli* strains with Diversilab rep-PCR method in Australia and determined that 15 of them were in the same clonal group. When they have compared the results of the *E.coli* isolates isolated during outbreaks in England and studied in the same way with the help of web, they have determined that they were the clones with similar profiles.

Pereira et al.^19^ have evaluated the clonal relationship of 144 ESBL-producing and quinolone-resistant *E.coli* strains that they have collected from 17 different centers in their study conducted in Brazil with the pulsed field gel electrophoresis (PFGE) method and 46 different patterns were identified. Although they have explained this situation with increased genetic diversity and the different resistance mechanisms due to half of the patients were from outpatient clinic andit was emphasized that there was a clonal relationship in the samples sent fromthe same center and this was an index for the transition between the patients. In our study, a total of 15 clones including 7 (A-G) and 8 sporadic cases were found according to rep-PCR results in 95 ESBL-producing and quinolone-resistant *E.coli* strains. This was explained with thegenetic diversity of *E.coli* and the existence of different mechanisms of resistance for the development of quinolone resistance. As in the study conducted by Pereira et al.^19^ despite most of our patients were outpatients and had community-acquired infections, this was not considered as the cause of genetic diversity. Because despite 65.3% of the strains in this study were isolated from CAI, clonal similarity with those isolated from the HAI, those with CAI had 4 risk factors as an average and 76.8% of them had at least one risk factor including urinary catheterization, prolonged hospitalization and undergoing surgeryin the last 6 months suggested that these strains may be hospital-acquired. This situation has one again demonstrated that the history of patients with prediagnosis of CAI should be evaluated better and these data should be recorded.

In our study, clone C was determined as the dominant clone. Although most of the strains belonging to A, C and D were isolated from CAI, these clones were considered to be associated with the hospital since they showed clonal proximity to those isolated from patients with HAI and the relationship of patients with hospital.

E, F and G clones were determined as hospital-acquired clones. They were not considered as outbreak due to E and F clones were isolated from pediatric patients and G clone was isolated from internal ICU with intervals. The reason for this may be the inpatients in this section are colonized by these clones in the hospital environment and these clones may cause infection by selecting as a result of antibiotic policy of these clinics. E and F clones were only seen in pediatric patients and at different services of pediatric department and this may be associated with the collection of pediatric clinics in a separate building from the main hospital.

## CONCLUSION

To prevent the production of ESBL, inappropriate and unnecessary use of antibiotics should be avoided, the resistance should be monitored by applying surveillance programs and infection control precautions should be followed carefully for reducing the spread of resistance. In addition to this infection measures, the development of an effective recording system which collects all information about patients by paying attention to the principles of ethics and protection of patient rights and the extraction of accurate epidemiological data are extremely important for the control of resistant bacterial infections in the community.
